# Detection of Single-Nucleotide Polymorphism on *uidA* Gene of *Escherichia coli* by a Multiplexed Electrochemical DNA Biosensor with Oligonucleotide-Incorporated Nonfouling Surface

**DOI:** 10.3390/s110808018

**Published:** 2011-08-15

**Authors:** Gang Liu, Ruojun Lao, Li Xu, Qin Xu, Lanying Li, Min Zhang, Hao Shen, Sanjay Mathur, Chunhai Fan, Shiping Song

**Affiliations:** 1 Shanghai Institute of Measurement and Testing Technology, Shanghai 201203, China; E-Mails: xul@simt.com.cn (L.X.); xuq@simt.com.cn (Q.X.); Lily@simt.com.cn (L.L.); Zhangm@simt.com.cn (M.Z.); 2 Laboratory of Physical Biology, Shanghai Institute of Applied Physics, Chinese Academy of Sciences, Shanghai 201800, China; E-Mails: roki_l@126.com (R.L.); fchh@sinap.ac.cn (C.F.); 3 Institute of Inorganic Chemistry, University of Cologne, Cologne 50939, Germany; E-Mails: hshen@uni-koeln.de (H.S.); sanjay.mathur@uni-koeln.de (S.M.)

**Keywords:** electrochemical biosensor, single-nucleotide polymorphism (SNP), nonfouling electrode surface, *Escherichia coli*

## Abstract

We report here a practical application of a multiplexed electrochemical DNA sensor for highly specific single-nucleotide polymorphism (SNP) detection. In this work, a 16-electrode array was applied with an oligonucleotide-incorporated nonfouling surfaces (ONS) on each electrode for the resistance of unspecific absorption. The fully matched target DNA templated the ligation between the capture probe assembled on gold electrodes and the tandem signal probe with a biotin moiety, which could be transduced to peroxidase-based catalyzed amperometric signals. A mutant site (T93G) in *uidA* gene of *E. coli* was analyzed in PCR amplicons. 10% percentage of single mismatched mutant gene was detected, which clearly proved the selectivity of the multiplexed electrochemical DNA biosensor when practically applied.

## 1. Introduction

Single nucleotide polymorphism (SNP) is a single base mutation in a DNA sequence, with a usual alternative of two possible nucleotides at one position. In all sequence variations [1] SNPs are most prevalent and stably inherited types, which are regarded as potent genetic markers and promising indicators for biomedical research, drug development, clinical diagnosis, and disease therapy [2–5].

Therefore, it has great value to develop SNP analysis methods with high sensitivity, specificity, and cost-effectivity to meet the demand of fast, practical analysis for genetic analysis of diseases and subtle genetic risk factors. So far several methods for genotyping SNPs have been reported based on a wide variety of specific probes and enzymes, including allele-specific oligonucleotide (ASO) hybridization [6], oligonucleotide ligation assays (OLA) [7–9] and primer extension assays [10], connected with the signal reading out on different physical platforms covering fluorescence [8,11], mass spectroscopy [12,13], electrochemistry [14,15], surface plasmon resonance [16] and so on.

Among all the available read-out mechanisms, the electrochemical DNA biosensor [17–19] is attracting considerable interest due to its high sensitivity, low cost, and use of a miniaturized and portable device [20]. However electrochemical DNA biosensors are still often hampered by unspecific adsorption [21] in complex matrix and real-world samples. Many efforts have been taken to improve the performance of the electrode surface as a key interface where the specific biological response and electron transferring happens [22,23].

Oligo(ethylene glycol) (OEG) is popularly employed to prepare nonfouling surfaces for its effect of protein-resistance. Inspired by OEG, our lab developed a highly selective oligonucleotide-incorporated nonfouling electrode surface (ONS) and successfully applied it to electrochemical DNA sensors. The ONS was constructed by self-assembling of OEG-terminated thiols (SH-OEG) and thiolated DNA probes (SH-DNA) together on to the electrode. When used for hybridization detection, the electrochemical biosensor was demonstrated to have nonfouling property and repel nonspecific adsorption of proteins, leading to an electrochemical DNA sensor that can effectively identify DNA targets with higher sequence specificity even in the presence of complicated biological fluids (e.g., human sera) [24].

More recently, we developed a ligase-based strategy [25] for SNP detection with this ONS electrochemical DNA sensor on a 16-electrode array as a proof-of-concept experiment. Benefited from the excellent specificity of the ONS electrode [26], the sensor was demonstrated to have higher differentiation ability toward SNPs on synthesis DNA than that with other electrode surface.

In this work, we aimed to optimize the biosensing principle and demonstrate the practicality of the ONS-based electrochemical DNA biosensor. We analyzed a SNP on β-glucuronidase gene (*uidA*) of *Escherichia coli* (*E. coli*) (T93G) which is well-known to be of great significance to distinguish the most harmful E. coli O157:H7 to prevent a possible life-threatening outbreak and to treat the patient pertinently.

## 2. Experimental Section

### 2.1. Materials

DNA oligonucleotides were purchased from Sangon Inc. (Shanghai, China) with their sequences listed in Table 1. For SNP detection, Two capture probes and a signal probe were designed as following: Capture probes-wild and capture-mutant (CP-W and CP-M) were 26-base sequences which were thiolated at their 5′ terminals with spacer consisted of 12 “T”s and a -(CH_2_)_6_-alkyl chain. They differed only at their 3′-terminal base. Signal probe (SP) had a biotin at its 3′ end as an affinity label and a phosphate group at its 5′ end for ligation.

Ethylene glycol-terminated thiol (HS-(CH_2_)_11_-EG_2_-OH, OEG) was purchased from Prochimia (Poland). Tris-(hydroxymethyl)aminomethane was from Cxbio Biotechnology Ltd. Ethylenediaminetetraacetic acid (EDTA), MCH, and tris(2-carboxyethyl) phosphine hydrochloride (TCEP) were purchased from Sigma-Aldrich (St. Louis, MO, USA). TMB substrate (TMB = 3, 3′, 5, 5′ tetramethylbenzidine; Neogen K-blue low activity substrate) was purchased from Neogen (U.S.). Avidin-HRP (horseradish peroxidase) was from Roche Diagnostics (Mannheim, Germany). The buffer solutions involved in this study were as follows: the DNA immobilization buffer was 10 mM Tris-HCl, 1 mM EDTA, 10 mM TCEP (pH 7.4), and 1 M NaCl. Enzyme was diluted to a 0.1 M PBS buffer with 0.5% casein (pH 7.2). All solutions were prepared with Milli-Q water (18 MΩ·cm resistivity) from a Millipore system.

### 2.2. Electrochemical Measurements

The 16-electrode sensor arrays were from GeneFluidics (Monterey Park, CA, USA), with 16 groups of electrodes consisting of a gold working electrode in the centre surrounded by a gold auxiliary electrode and a gold reference electrode. Cyclic voltammetric (CV) and amperometric (I-t) measurements of DNA arrays were performed at room temperature using a 16-channel PM3000 workstation (GeneFluidics). In I-t measurements, the voltage was fixed at −100 mV (*versus* the gold reference electrode), and the reduction current was measured at 60 s.

### 2.3. Construction of the ONS at Gold Electrode Surfaces

5 μM thiolated capture probes in DNA immobilization buffer (3 μL) was added to each working electrode for 1 h at room temperature. Then electrodes were incubated with 2 mM OEG for 2 h to obtain mixed SAMs. The electrode was then rinsed with Milli-Q water and dried with N_2_ after each step.

### 2.4. PCR Amplification

The PCR amplification of genomic DNA fragments (*uidA* gene) was performed in a Bio-Rad PCR cycler (PTC-100). A pair of asymmetric primers (primer 1/primer 2 = 100:1) was employed in order to generate the ssDNA target. Of note, the use of asymmetric PCR ensured that the product was in the single-stranded format that could be directly sensed by the single stranded probe. The amplification was performed in 20 μL PCR buffer containing 2.0 units of Taq DNA Polymerase (Invitrogen). Thermocyler condition was 4 min at 94 °C 30 cycles of 55 s at 94 °C, 60 s at 60 °C, 60 s at 72 °C, and then a final extension at 72 °C for 5 min. PCR fragment size (250 bp) was confirmed by agarose gel electrophoresis.

### 2.5. SNP Detection

One hundred μL mixed solutions containing the proper concentration of synthesized DNA target (or 20 μL PCR product), 100 nM signal probe, 2 U ampligase (Epicentre), and 0.5% BSA were preannealed at 50 °C for 5 min and then added to each working electrode with mixed SAMs. As the employed ampligase is a relatively thermostable ligase, the ligation was performed at 55 °C. After 20 min incubation, a denaturation step was followed by treating with 100 mM NaOH at 70 °C for 10 min. After being washed, electrodes were treated with 1% BSA and then the avidin-HPR (0.5 U/mL in 0.1 M PBS buffer with 0.5% casein) was added. After 15-min incubation at room temperature, electrodes were washed with 0.1 M NaCl and 10 mM PBS buffer (pH 7.4) and subjected to electrochemical measurements.

## 3. Results and Discussion

**Scheme 1.**
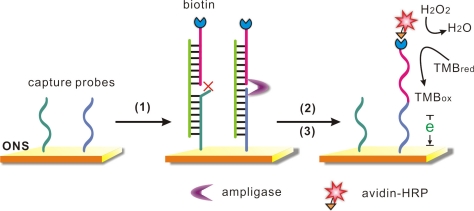
Schematic illustration of the ligation based electrochemical DNA biosensor for the SNP analysis on an ONS electrode. (1) Target DNA (or PCR amplicons) and ligase; (2) Washing step; (3) Avidin-HRP. Capture probes assembled on the ONS electrode had different end bases at 3′ terminals, only when the target DNA could perfectly hybridize to the capture probes and form a “sandwich” structure with the signal probes, the ligation between two probes could be performed and let the signal probes survive in the following washing step. The avidin-HRP was then combined by the biotin label and catalyzed the redox reaction of the TMB substrate.

Our SNP detection strategy was based on the DNA-templated ligation and the outstanding property of the ONS electrode (Scheme 1). Gold electrodes were first covered with the capture probes which combined to the gold surface through strong Au-S covalent bonds, and then SH-OEG was added to form a SH-DNA (capture probes)/SH-OEG self-assembled monolayer (SAM). Two capture probes (capture wild/mutant, CP-W/M) were employed in this work, which differed only at the last base at 3′-terminal as listed in Table 1. A “sandwich” like structure would be formed in the presence of a target DNA and a signal probe. Depending on the last base, each capture probe could only hybridize to one target DNA with perfect matching (CP-W to target W, CP-M to T-M), but had a missmatch point adjacent to the signal probe with the other DNA (CP-W to T-M, CP-M to T-W). When the specific hybridization was fully matched, a DNA templated ligation would be performed by the function of the ligase, as a result, the signal probe with a biotin label covalently bound to the capture probe. In contrast, the ligation would be stopped due to the mismatched adjacent point, and then a stringent wash step would denature the sandwich structure and remove the unbound signal probe. After the ligation and the following wash step, avidin-HRP is incubated with the surface, which would catalyze the reduction reaction of hydrogen peroxide and provide amperometric readout for the SNP detection. In order to realize simultaneous detection of two possible alleles, we employed a 16-electrode sensor array to perform multiplexed SNP genotyping.

With the purpose of verification and optimization of the anlaysis strategy, we synthesized two 26-base DNA targets (T-W and T-M), that had sequences corresponding to a fragment of the wild and mutant type *uidA* gene respectively, which means the T-W fully match with CP-W, but had a single mismatch point with CP-M; the T-M did just the opposite.

**Figure 1.**
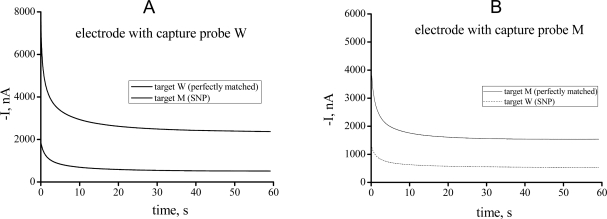
Amperometric curves for the detection of single base mismatched (dashed line) and complemental probe (solid line) in the presence of the complementary target of 5 nM. **(A)** Analysis performed on electrodes with CP-W, where the T-W was fully matched and generated an enhanced current signal; **(B)** Analysis on electrode with CP-M, where T-M produced a higher signal than T-W. Potential of amperometry: 0.1 V.

When the target DNA was added, it hybridized to the capture probe and combined a signal probe to form a “sandwich” structure. If fully complementary to the capture probe, the target DNA would template a ligation between two probes with the effect of a ligase, leading to a significantly enhanced HRP-catalyzed reduction current. When there was a mismatched site, the reduction current signal was decreased due to the absence of ligation and wash-off of the signal probe. The amperometric I-t curves provide quantitative measurements, which reveal that the differentiation ratio between the fully complementary target and the mutant was as high as 4-fold (Figure 1). We thus demonstrated that, by using appropriate probes and ligase, unambiguous electrochemical signals could be achieved for the fully matched DNA while not the mismatched sequence harboring the SNP.

**Figure 2.**
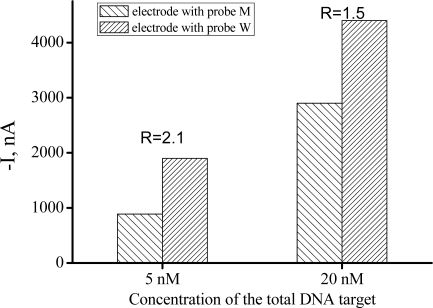
Optimization of the total target concentration. The DNA target sample consisted of 10% mutant DNA (T-M) and 90% wild DNA (T-W).

For the analysis of a mixed target solution, the concentration of the total DNA target is critical for the performance of the biosensor. As shown in Figure 2, DNA samples containing 10% mutant DNA (T-M) and 90% wild DNA (T-W) was analyzed by two different electrodes, the current signals were all increased on both electrodes when the total concentration was increased from 5 nM to 20 nM, but the signal difference ratio R (Ratio of current signal from electrode with CP-W to that from electrode with CP-M) was decreased (from 1.5 to 2.1). It’s easy to understand, the fully matched signal would obviously increase with the increase of the total concentration; on the other side, more mismatched target would also generate higher unspecific adsorption, more inaccurate ligation and more survival in denaturation step which would hamper the discrimination of the biosensor. So we choose 5 nM as an optimized total target concentration for the analysis of a mixed DNA sample.

**Figure 3.**
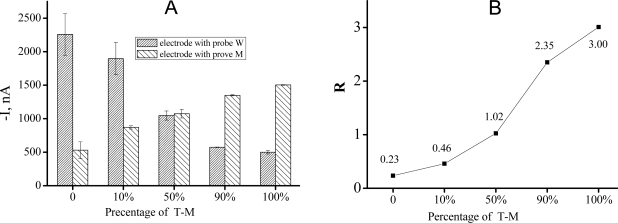
Results of a multiplexed SNP analysis on a 16-electrode array. The mixed sample consisted of T-M and T-W with a serious concentration ratio. **(A)** Comparison of current signals from two kinds of electrode when the concentration ratio was changed; **(B)** The signal difference ratio (R) got higher while the percentage of T-M was increased. The total concentration of target DNA was 5 nM.

We then employed a 16-electrode sensor array to perform multiplexed SNP detection of a series of samples with different concentration ratio of T-W and T-M. Firstly two capture probes (CP-W/M) were assembled onto the electrodes. When the mixed sample was added, only the perfectly matched targets would template the ligation and consequently cause a catalyzed current signal while mismatched ones could not. As shown in Figure 3, the average signals of I-t curves from different electrodes were changed highly depended on the concentration ratio of mismatched target: When the percentage of the T-M increased from 0% to 100%, the current signal from electrode with CP-W decreased while that from electrode with CP-M increased. When we calculated the signal difference ratio R (Figure 3(B)), it was more than 10 times higher at 100% mutation ratio than at 0%, and 10% mutant DNA (T-M) was clearly discriminated based on the signal ratio.

**Figure 4.**
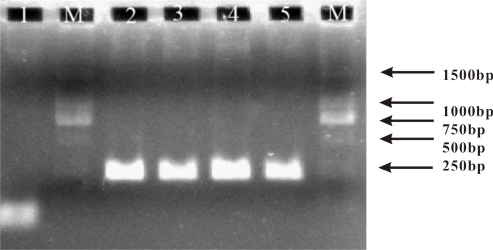
Electrophoretic analysis of the PCR amplicons for the *uidA* gene of *E. coli*. The box indicates the 250-bp product (*uidA*). 1, Negative control (NC); M, DNA size marker; 2, wild type *uidA* gene; 3, mutant type *uidA* gene; 4 mixed sample with 10% wild type *uidA* gene; 5, mixed sample with 50% wild type *uidA* gene.

Finally, to challenge the practical applicability of our multiplexed electrochemical biosensor, we analyzed a PCR amplicons from mixed sample containing both the wild and mutant *uidA* gene of *E. coli*. By using an asymmetric PCR protocol [27,28], we obtained ssDNA targets of 250 bp that could be directly sensed. As shown by the result of electrophoretic analysis (Figure 4), PCR amplification got anticipative products from all samples including wild type *uidA* gene, mutant type *uidA* gene, and their mixture.

**Figure 5.**
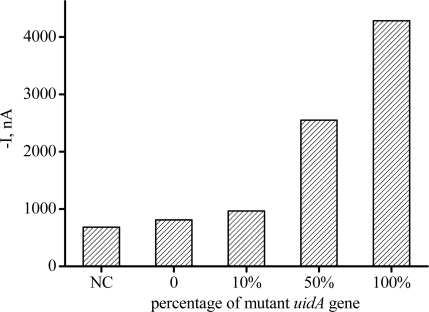
the result of the multiplexed electrochemical analysis of PCR amplicons from samples with different percentage of mutant *uidA* gene.

Amplicons from different templates were analyzed by our biosensor of electrodes with capture-M, the result was shown in Figure 5. Interestingly, our sensor performed equally well despite the fact that this target is much longer than the model. The negative control (NC) and mismatched sample (100% wild *uidA* amplicon) got only negligible signal. But when the percentage of mutant *uidA* template increased, the amperometric signal accordingly got higher. We found that, by coupling asymmetric PCR, this sensor could get as high as about five-fold signal distinction between 100% mutant and 100% wild wild *uidA* PCR amplicons. By this multiplexed electrochemical biosensor 10% mutant *uidA* genomic DNA could be selectively identified.

## 4. Conclusions

In summary, we have optimized the performance of the ONS- and ligation-based electrochemical DNA sensor for its practicability, and utilized the method for the detection of a mutant site (T93G) in *uidA* gene of *E. coli* which is known to be an important marker for the bacteria hazard analysis. We demonstrate that the 16-electrode sensor array provides a multiplexed platform for simultaneous detection of allelic genes, and 10% mismatched DNA is successfully distinguished. Finally, a series of PCR amplicons from templates of increasing percentage of mutant DNA is analyzed. Results showed that the signal distinction was as good even when the target sequence is lengthened to 250 bp in a complex PCR product. Therefore, we proved the ONS electrochemical DNA biosensor to be a promising tool for the selective and inexpensive analysis of SNPs in practical application.

## Figures and Tables

**Table 1.** Sequences of oligonucleotides.

Capture probe-wild (CP-W)	5′-SH-TTTTTTTTTTTTCCCACCAACGCTGA-3′
Capture probe-mutant (CP-M)	5′-SH-TTTTTTTTTTTTCCCACCAACGCTGC-3′
Signal probe (SP)	5′-TCAATTCCACAGTTTTCGCGTTTTT-biotin-3′
Target-wild (T-W)	5′-CTGTGGAATTGATCAGCGTTGGTGGG-3′
Target-mutant (T-M)	5′-CTGTGGAATTGAGCAGCGTTGGTGGG-3′

## References

[1] Vignal A, Milan D, SanCristobal M, Eggen A (2002). A review on SNP and other types of molecular markers and their use in animal genetics. Genet. Sel. Evol.

[2] Frazer KA, Ballinger DG, Cox DR, Hinds DA, Stuve LL, Gibbs RA, Belmont JW, Boudreau A, Hardenbol P, Leal SM (2007). A second generation human haplotype map of over 3.1 million SNPs. Nature.

[3] Scuteri A, Sanna S, Chen WM, Uda M, Albai G, Strait J, Najjar S, Nagaraja R, Orru M, Usala G (2007). Genome-wide association scan shows genetic variants in the FTO gene are associated with obesity-related traits. PLoS Genet.

[4] He Y, Zeng K, Gurung AS, Baloda M, Xu H, Zhang X, Liu G (2010). Visual detection of single-nucleotide polymorphism with hairpin oligonucleotide-functionalized gold nanoparticles. Anal. Chem.

[5] Shabo A (2008). Integrating genomics into clinical practice: Standards and regulatory challenges. Curr. Opin. Mol. Ther.

[6] Iannone MA, Taylor JD, Chen J, Li MS, Rivers P, Slentz-Kesler KA, Weiner MP (2000). Multiplexed single nucleotide polymorphism genotyping by oligonucleotide ligation and flow cytometry. Cytometry.

[7] Taton TA, Mirkin CA, Letsinger RL (2000). Scanometric DNA array detection with nanoparticle probes. Science.

[8] Duan X, Liu L, Wang S (2009). Homogeneous and one-step fluorescent allele-specific PCR for SNP genotyping assays using conjugated polyelectrolytes. Biosens. Bioelectron.

[9] Gaunt TR, Hinks LJ, Rassoulian H, Day IN (2003). Manual 768 or 384 well microplate gel “dry” electrophoresis for PCR checking and SNP genotyping. Nucleic Acids Res.

[10] Pastinen T, Raitio M, Lindroos K, Tainola P, Peltonen L, Syvanen AC (2000). A system for specific, high-throughput genotyping by allele-specific primer extension on microarrays. Genome Res.

[11] Okamoto A, Ichiba T, Saito I (2004). Pyrene-labeled oligodeoxynucleotide probe for detecting base insertion by excimer fluorescence emission. J. Am. Chem. Soc.

[12] Fei Z, Smith LM (2000). Analysis of single nucleotide polymorphisms by primer extension and matrix-assisted laser desorption/ionization time-of-flight mass spectrometry. Rapid Commun. Mass Spectrom.

[13] Tost J, Gut IG (2002). Genotyping single nucleotide polymorphisms by mass spectrometry. Mass Spectrom. Rev.

[14] Zhang S, Wu Z, Shen G, Yu R (2009). A label-free strategy for SNP detection with high fidelity and sensitivity based on ligation-rolling circle amplification and intercalating of methylene blue. Biosens. Bioelectron.

[15] Di Giusto DA, Wlassoff WA, Giesebrecht S, Gooding JJ, King GC (2004). Multipotential electrochemical detection of primer extension reactions on DNA self-assembled monolayers. J. Am. Chem. Soc.

[16] Sipova H, Springer T, Homola J (2011). Streptavidin-enhanced assay for sensitive and specific detection of single nucleotide polymorphism in TP53. Anal. Bioanal. Chem.

[17] Fan C, Plaxco KW, Heeger AJ (2003). Electrochemical interrogation of conformational changes as a reagentless method for the sequence-specific detection of DNA. Proc. Natl. Acad. Sci. USA.

[18] Zhang J, Song S, Zhang L, Wang L, Wu H, Pan D, Fan C (2006). Sequence-specific detection of femtomolar DNA via a chronocoulometric DNA sensor (CDS): Effects of nanoparticle-mediated amplification and nanoscale control of DNA assembly at electrodes. J. Am. Chem. Soc.

[19] Zhang J, Song S, Wang L, Pan D, Fan C (2007). A gold nanoparticle-based chronocoulometric DNA sensor for amplified detection of DNA. Nat. Protoc.

[20] Akagi Y, Makimura M, Yokoyama Y, Fukazawa M, Fujiki S, Kadosaki M, Tanino K (2006). Development of a ligation-based impedimetric DNA sensor for single-nucleotide polymorphism associated with metabolic syndrome. Electrochim. Acta.

[21] Li D, Song S, Fan C (2010). Target-responsive structural switching for nucleic acid-based sensors. Acc. Chem. Res.

[22] Liu G, Wan Y, Gau V, Zhang J, Wang L, Song S, Fan C (2008). An enzyme-based E-DNA sensor for sequence-specific detection of femtomolar DNA targets. J. Am. Chem. Soc.

[23] Song S, Qin Y, He Y, Huang Q, Fan C, Chen H-Y (2010). Functional nanoprobes for ultrasensitive detection of biomolecules. Chem. Soc. Rev.

[24] Zhang J, Lao R, Song S, Yan Z, Fan C (2008). Design of an oligonucleotide-incorporated nonfouling surface and its application in electrochemical DNA sensors for highly sensitive and sequence-specific detection of target DNA. Anal. Chem.

[25] Wan Y, Zhang J, Liu G, Pan D, Wang L, Song S, Fan C (2009). Ligase-based multiple DNA analysis by using an electrochemical sensor array. Biosens. Bioelectron.

[26] Wan Y, Lao R, Liu G, Song S, Wang L, Li D, Fan C (2010). Multiplexed electrochemical DNA sensor for single-nucleotide polymorphism typing by using oligonucleotide-incorporated nonfouling surfaces. J. Phys. Chem. B.

[27] Lai RY, Lagally ET, Lee SH, Soh HT, Plaxco KW, Heeger AJ (2006). Rapid, sequence-specific detection of unpurified PCR amplicons via a reusable, electrochemical sensor. Proc. Natl. Acad. Sci. USA.

[28] Poddar SK (2000). Symmetric *vs*. asymmetric PCR and molecular beacon probe in the detection of a target gene of adenovirus. Mol. Cell. Probes.

